# Improvement on Esmarch’s Elastic Bandage

**Published:** 1873-11

**Authors:** W. Harrison Cripps


An Improvement on Esmarch's Elastic Bandage.— *W. Harrison Cripps.* — Esmarch's admirable suggestion of using an elastic bandage to exclude the blood before operating on limbs, and the complete success attending it, are now probably well known. The following is a simple modification of his arrangement, by which many yards of elastic bandage may be dispensed with, and it can be easily and quickly applied.

A short india-rubber tube is used, not only to prevent the blood from returning to the limb, but also for the purpose of removing it in the first place. The two ends of an india-rubber tube, twenty-one inches in length, and about three-eighths of an inch thick, are bound together with a piece of twine, the whole forming an elastic ring seven inches in diameter. A grooved reel revolving between a double handle completes the necessary apparatus.

To apply this to the arm, three or four complete turns of the elastic ring are wound tightly round the hand in such a manner as to include the fingers and thumb, care being taken that the turns lie even and not across one another. The reel is then put under the free portion of the ring connecting the upper and lower coil. The reel is passed round and round the limb in an upward direction; thus each coil is unwound from below as another is added above. In this way four tight coils of india-rubber are carried up the limb to any distance required. The degree of tightness can be regulated with the greatest nicety by the distance the reel is drawn from the limb by the bandager.

This method of driving blood from the limb answers perfectly in the arm, and in the lower part of the leg; but in carrying the bandage over the popliteal space the flexor tendons prevent the artery being effectually compressed. A firm pad in the space would probably answer the purpose.

To remove the bandage, it may either be unrolled by reversing the action of the reel, or the twine connecting its two ends may be cut with scissors.

The Physiology of Menstruation.—In inquiring now into the physiological relations of the three processes—the swelling of the mucosa, the discharge of the ovum, and the flow of menstrual blood—Kundrat insists strongly upon the ascertained chronology of the events. The first mentioned of the three is the first in order of time, and it is almost certainly the preparation for the reception of the ovum· It is much more improbable that the uterus during the menstrual flow is in a condition suitable for this function—with a retrogressive process going on in the mucosa, its vessels ruptured, and its surface discharging blood. It is even more improbable that the mucosa in this state of degeneration will on the descent of an ovum take on a totally opposite process, and become highly developed. The type of the impregnated uterus is seen in the active uterus when the mucosa is swollen and mensturation has not yet commenced. If the bleeding does not commence, it is a sign that the ovum has perished, and that the mucosa is returning to a state of rest. Thus we arrive at the highly-important conclusion that a developing ovum, or growing embryo, belongs not to a menstrual period just passed, but to one just prevented by fecundation. Lowenhorst has already expressed this opinion from a consideration of the clinical aspects of menstruation, and we believe that the method of calculating the duration of pregnancy suggested by the new facts is not altogether a new one among the gynæcologists and practitioners of this country.

A Cremation Club.—The *Lancet* learns that a club has been formed at Hamburg for promoting the practice of incineration instead of burial. The club is said to number eighty members, each of whom on entering makes a will directing the disposal of his body after death.

